# Reciprocal information flow and role distribution support joint action coordination

**DOI:** 10.1016/j.cognition.2019.02.006

**Published:** 2019-06

**Authors:** Arianna Curioni, Cordula Vesper, Günther Knoblich, Natalie Sebanz

**Affiliations:** a Department of Cognitive Science, Central European University, Budapest, Hungary; b School of Communication and Culture, Aarhus University, Aarhus, Denmark

**Keywords:** Joint action, Internal models, Role distribution, Coordination

## Abstract

Many joint actions require task partners to temporally coordinate actions that follow different spatial patterns. This creates the need to find trade-offs between temporal coordination and spatial alignment. To study coordination under incongruent spatial and temporal demands, we devised a novel coordination task that required task partners to synchronize their actions while tracing different shapes that implied conflicting velocity profiles. In three experiments, we investigated whether coordination under incongruent demands is best achieved through mutually coupled predictions or through a clear role distribution with only one task partner adjusting to the other. Participants solved the task of trading off spatial and temporal coordination demands equally well when mutually perceiving each other’s actions without any role distribution, and when acting in a leader-follower configuration where the leader was unable to see the follower’s actions. Coordination was significantly worse when task partners who had been assigned roles could see each other’s actions. These findings make three contributions to our understanding of coordination mechanisms in joint action. First, they show that mutual prediction facilitates coordination under incongruent demands, demonstrating the importance of coupled predictive models in a wide range of coordination contexts. Second, they show that mutual alignment of velocity profiles in the absence of a leader-follower dynamic is more wide-spread than previously thought. Finally, they show that role distribution can result in equally effective coordination as mutual prediction without role assignment, provided that the role distribution is not arbitrarily imposed but determined by (lack of) perceptual access to a partner’s actions.

## 1 Introduction

Being able to coordinate our actions with others is one of the most remarkable human social abilities. Playing ensemble music, working in a team of surgeons, or even just passing items from the shopping bag to someone filling the fridge involves not only a general willingness to cooperate, but fine-grained temporal and spatial coordination. How do people succeed in coordinating their actions with each other? Is it important that both task partners adjust to each other, or can a clear distribution of leader and follower roles lead to similar or even better coordination?

Previous research indicates that interaction partners’ ability to mutually adapt to each other and to engage in predictions about each other’s actions plays an important role in achieving joint action coordination (e.g., Knoblich and Jordan, 2003, Keller et al., 2007, Kourtis et al., 2013, Vuust and Witek, 2014; see Keller, Novembre, & Hove, 2014 for a review). For example, Konvalinka, Vuust, Roepstorff, and Frith (2010) compared performance in a joint tapping task where two individuals could mutually hear each other to a condition where only one of them could hear and therefore adapt to the other. In the reciprocal condition, both participants contributed to the coordination, with each of them adjusting the timing of their next tap based on the other’s previous tap. Synchronization performance was better when both participants received information about each other’s actions than when the unidirectional flow of information turned one of them into a leader and the other into a follower.

Interestingly, coordination can suffer from an explicit role distribution into leader and follower even when two task partners can mutually perceive each other’s actions (Noy, Dekel, & Alon, 2011). Noy and colleagues asked improvisation experts to perform a simplified version of the “mirror game”, a theatre improvisation exercise that requires performing the same, improvised actions in synchrony. In this task, two participants facing each other each moved a slider along a track, under the instruction to create synchronized and interesting motions together. The authors compared synchronization in a condition where one person was designated as the leader to a condition where the two improvisation experts were instructed to simply move together. Having no designated leader resulted in better coordinated velocity profiles across the two members of a pair.

The improved coordination performance in the context of reciprocal information flow can be explained by the notion of coupled forward models (Noy et al., 2011). Synchronization involves predicting others’ actions (Keller et al., 2007, Ramenzoni et al., 2015), which can be achieved by relying on predictive models in one’s own motor system (Wolpert et al., 2003, Franklin and Wolpert, 2011). When two interaction partners mutually predict each other’s actions, the output of one system provides the input for the other, resulting in the coupling of the partners’ predictive models. This could allow for more precise coordination than when only one individual engages in predictions about the other. It is important to note that for rhythmic coordination tasks, unidirectional informational couplings could account for at least part of the stability of the coordination (Richardson, Campbell, & Schmidt, 2009).

However, in contrast to the findings of the previously reported studies, there is also evidence suggesting that instructed or emerging role distributions can be beneficial for joint action coordination. Noy et al. (2011) found that, contrary to improvisation experts, novices achieved better coordination in the mirror game when one of them was instructed to lead and the other to follow, compared to no such role assignment, highlighting the role of expertise in joint action. An example for how an emergent role distribution into leader and follower (Roberts and Goldstone, 2011, Skewes et al., 2015) enables coordination is provided by a recent study by Richardson et al. (2015). They devised a joint action task that required two participants to move stimuli back and forth on crossing paths without colliding. The task was designed such that if both participants moved in synchrony and followed a straight path, they would bump into each other. Nearly all pairs converged on an effective solution where one partner in a pair followed an elliptical trajectory while the other followed a straight path. The partner following the elliptical trajectory was more influenced by the movements of the partner following a straight line than vice versa, indicating that a leader-follower dynamic emerged. This emergent role distribution may be beneficial for coordination because the leader provides a stable and predictable input to the partner who is in charge of compensating and adapting. Indeed, making one’s own actions predictable has been identified as a useful coordination strategy in joint tasks (Vesper et al., 2011, Vesper et al., 2016).

To sum up, on the one hand, some findings suggest that a leader-follower dynamic can be detrimental for interpersonal coordination; such a detriment can be caused by environmental constraints that do not allow for reciprocal information flow (Konvalinka et al., 2010) or by an assigned role distribution that keeps one individual from adjusting to and predicting the other’s actions (Noy et al., 2011). On the other hand, other findings indicate that effective role distributions in terms of a leader and follower emerge when some role differentiation is clearly required (Richardson et al., 2015). This raises the question under which conditions role distribution emerges when it is not strictly required by the coordination task, and what makes role distribution beneficial or detrimental to joint action coordination.

The current study aims at providing answers to these questions. Our starting point is the important observation that the coordination tasks used in previous studies differ in one key aspect: the congruency of temporal and spatial coordination demands. The joint tapping task used by Konvalinka et al. required only temporal coordination; in the mirror game employed by Noy et al. achieving temporal coordination implied achieving spatial coordination and vice versa, once aligned on a starting position. Accordingly, these tasks involved *congruent coordination demands* because there was no trade-off between achieving temporal and spatial coordination. In contrast, the collision avoidance task devised by Richardson et al. involved *incongruent coordination demands* because it implied a trade-off between achieving spatial and temporal coordination. This raises the possibility that role distributions have different effects depending on the demands a joint task imposes on spatial and temporal coordination. Accordingly, the first aim of the present study was to determine how role distribution affects coordination under congruent and incongruent coordination demands. A second and related aim was to determine whether coordination benefits from assignment of roles when reciprocal information flow between joint action partners is available.

**Fig. 1:**
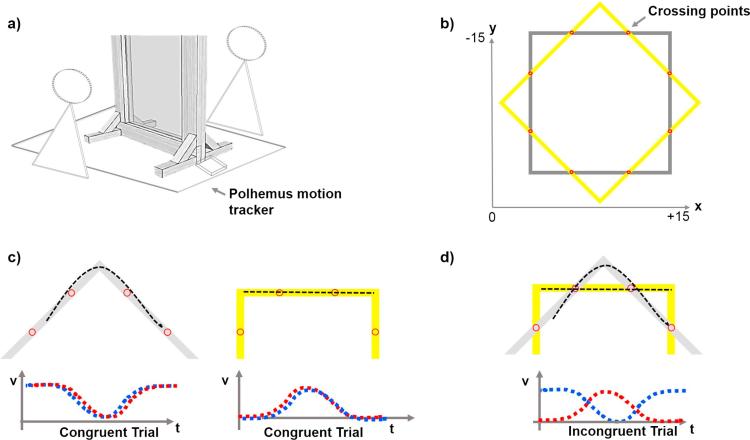
Panel (a): Experimental set-up. Two participants were seated opposite each other. Their task was to trace the shapes drawn on the glass panel in between them using their right index finger. Panel (b): Two squares of different colours were positioned on top of each other with a 45° rotation on the centroid. On congruent trials, the two participants in a pair traced the same shape. On incongruent trials, they each traced a different shape. They were instructed to pass the crossing points as synchronously as possible. These crossing points were used to calculate participants' coordination performance. Panel (c): schematic representation of the expected velocity profiles for congruent trials (upper row: shape segments; lower row: velocity profile in red for one participant and in blue for the other). When participants traced a corner of the shape (c, left graph), we expected their movement velocity to drop as the curvature is high; when participants traced straight-line segments (c, right graph) we expected a bell-shaped velocity profile (Flash & Hogan, 1985). Panel (d) schematic representation of the expected velocity profiles for incongruent trials: the velocity profiles of two participants are incongruent, as they are tracing a straight-line and a corner segment at the same time.

**Fig. 2:**
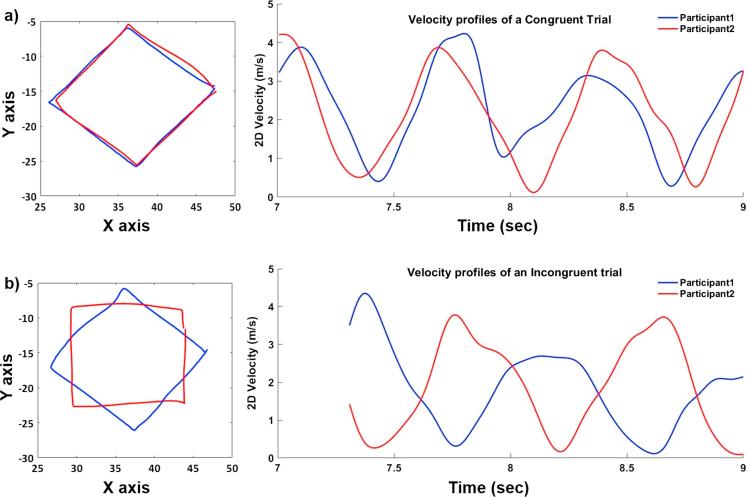
Example of tracing trajectories and velocity profiles for a congruent (a) and an incongruent trial (b). Panel b shows the velocity profiles of two participants while tracing half of the shape, which corresponds to approximately 2 s.

We developed a new task that required pairs of participants to synchronize their arrival times at pre-defined coordination points while tracing the same shape or different shapes (see Fig. 1). We chose this continuous coordination task because continuous visuo-motor performance relies on internal predictive models of individual trajectories to maximize control and compensate for sensorimotor delays (Neilson et al., 1988, Kawato, 1999, Franklin and Wolpert, 2011). In order to manipulate coordination demands, we exploited the natural variation of movement speed in tracing rectangular shapes. In particular, straight lines are normally performed with a velocity peak around the centre of the line, whereas moving past corners requires a reduction of velocity in order to change movement direction. In our coordination task, tracing the same shape implied *congruent coordination demands* for the partners of a pair because there was no trade-off between achieving spatial and temporal coordination (see Fig. 1, Panel c). Specifically, synchronously slowing down before corners and speeding up after corners was a valid strategy to achieve coordination on both dimensions. In contrast, tracing different shapes implied *incongruent coordination demands* because there was a trade-off between achieving spatial and temporal coordination (see Fig. 1, Panel d). The coordination points were placed in regions where one partner needed to slow down because she approached a corner whereas the other partner needed to speed up on a straight segment to arrive at the next coordination point in time. In this situation, maintaining speed to achieve accurate temporal coordination reduced spatial accuracy because accurately tracing corners requires zero velocity at turning points. To achieve coordination in the face of incongruent coordination demands, the two partners in a pair either needed to sacrifice spatial accuracy at corners (where more curved trajectories allow for maintaining a higher speed) or temporal accuracy (implying increased asynchronies at coordination points) (see Fig. 2).

In Experiment 1, we compared joint action coordination with congruent and incongruent coordination demands when no role distribution was prescribed and when partners could mutually perceive each other’s actions (‘Reciprocal information flow without role assignment’). In Experiment 2 (‘Reciprocal information flow with role assignment’) we investigated whether and how coordination benefits or suffers from assigning the role of leader to one partner and the role of follower to the other partner. In Experiment 3 (‘Unidirectional information flow with role assignment’) we investigated whether any coordination costs or benefits of assigning roles also occur under conditions of unidirectional information flow where one partner can perceive the actions of the other but not vice versa. If establishing a task distribution by assigning leader-follower roles impairs coordination, we should see worse coordination in Experiment 2 than in Experiment 1. If reciprocal information flow facilitates coordination, partners should achieve better spatial and temporal coordination in Experiment 1 and 2 than in Experiment 3.

## 2 Experiment 1 – reciprocal information flow without role assignment

In Experiment 1, participants performed the joint tracing task without designated roles. Assuming that coordination is easier when spatial and temporal coordination demands are congruent and harder when they are incongruent and require a trade-off, we predicted higher spatial accuracy and temporal synchronization at pre-defined crossing points on congruent than on incongruent trials. We also predicted that participants would be able to coordinate their actions on incongruent trials at least to some extent by adapting the velocity of their movements (in particular, slowing down while moving along a straight line while their partner is tracing a corner) and/or by modulating spatial movement parameters (in particular, increasing the curvature on corners, as “cutting corners” should make it easier to align with the partner concurrently tracing a straight line). If reciprocal information about each other’s actions allows interaction partners to increase the accuracy of their predictions about the partner’s movements over time, as suggested by the notion of coupled forward models, we should also observe improvements in synchronization performance across the joint task performance, both on congruent and on incongruent trials.

### 2.1 Methods

#### 2.1.1 Participants

Ten randomly composed pairs of right-handed individuals (11 female, average age = 23.6 years, SD age = 1.93 years) participated in the study. The members in each pair did not know each other prior to participation. They signed prior informed consent and received monetary compensation. The study was performed in accordance with the Declaration of Helsinki.

#### 2.1.2 Apparatus

The two participants were seated opposite each other at a table (80 × 80 cm). A 50 × 50 cm glass panel sustained by a wooden frame (54 × 54 cm) was placed along the midline of the table, equidistant from both participants (40 cm) (Fig. 1a). Two 15 × 15 cm squares (5 mm line width) of different colours (yellow and white) were positioned on top of each other with a 45° rotation on the centroid and centred along the midline of the glass panel (25 cm) (Fig. 1b). A set of 6 LED lamps was fixed on top of the frame and directed perpendicularly to the glass, to ensure the shapes were equally visible from both sides. A 50 × 22 cm black fabric was used to cover the upper part of the glass panel so that participants could not see each other’s faces.

A Polhemus G4 electro-magnetic motion capture system (40 Hercules Drive, Colchester, Vermont) was used to record participants’ movement data at a constant sampling rate of 120 Hz (approximately a frame of three dimensional Cartesian coordinates every 8 ms). For that purpose, a motion capture micro-sensor (1.8 mm) was attached to the front of the nail of the outstretched index finger of each participant’s right hand. The experimental procedure and the data recording was controlled on line with MATLAB (2015b). All material used in the experimental set up was metal-free to avoid interference with the kinematic recordings.

#### 2.1.3 Procedure

At the beginning of the experiment both participants received written instructions about the task and the experimental procedure. Both participants individually performed a calibration and a practice part before they started to perform the joint task.

##### 2.1.3.1 Individual calibration

During calibration we recorded 16 reference coordinates, corresponding to the four corners of each of the two superimposed shapes, and the eight crossing points where the two shapes overlapped (Fig. 1b). Each participant was asked to position her right index finger on each of the 16 calibration points one by one. When the participant positioned the index finger correctly, the experimenter initiated a 1 sec recording of the position. The averaged 3D positions of each calibration point of each participant were used for online control of the trial procedure and for offline data analysis. The same calibration routine was repeated at the end of the experiment to control for distortions and measurement errors.

##### 2.1.3.2 Individual practice

After calibration, all participants performed an individual practice aimed at establishing a movement tempo of 45 bpm. The practice consisted of eight trials. In each trial participants traced one of the two shapes four times without interrupting their movement. A beeping sound set to the tempo indicated the pace of the movement (∼1.3 s from corner to corner). Participants were asked to pay attention to the tempo and to keep this tempo during individual practice as well as later during joint performing. Trial by trial feedback was provided based on the overall duration of the tracing movement in a trial (target duration based on the frequency of the beat = 20.8 s; duration < 15 sec = “too fast”, duration > 25 sec = “too slow”).

##### 2.1.3.3 Joint task

Participants were asked to use their right index finger to trace the shapes on the glass panel. Both participants in a pair received the same task instructions: “Trace your shape keeping the tempo you trained in the individual practice and be as spatially accurate as possible without stopping. Your task is to be coordinated with your partner, i.e., to meet your partner at the crossing points of the two shapes. At the beginning of each trial you will hear “yellow” or “white”, instructing you to trace the yellow or white shape. Your partner will also hear “yellow” or “white” and will trace the corresponding shape. In some trials you will trace the same shape, in some trials each of you will trace a different shape.”

Participants started with their hands in a resting position along the midline of the table. The experimenter manually started each trial and participants received instructions for the following trial (‘white’ or ‘yellow’). Then participants placed their hands in their respective starting positions on opposite sides of the glass panel. Starting positions in all conditions were 7.5 cm apart. One of the participants (Participant 1) was instructed to start moving as soon as a go signal occurred (tone of 100 ms duration, 880 Hz) and the other participant (Participant 2) was instructed to start when Participant 1 reached the midline of the first segment of the shape. This allowed Participant 1 to start moving at the practiced movement speed. Participants were instructed to continue tracing the shape until they received a stop signal (100 ms duration, 660 Hz). Each trial consisted of four rounds of tracing. Unfiltered movement data was evaluated online to determine when both participants had completed four rounds of tracing. To that end, a MATLAB algorithm was used to track participants’ position online. The stop signal was automatically delivered as soon as the coordinates of both participants’ sensors matched a reference coordinate four times. The whole experiment consisted of 4 blocks of 12 trials each, divided by short breaks to avoid fatigue. The congruency of the two shapes traced by the two participants (same or different shapes traced) was randomized within blocks. The experiment lasted about 1 h.

#### 2.1.4 Data analysis

We segmented participants’ movement data using the reference points collected during individual calibration. The eight coordinates where the two shapes intersected (crossing points) were used to measure absolute interpersonal asynchrony, spatial deviation, and movement velocity (see below).

***Absolute Asynchrony (ms)*** served as a measure of temporal coordination between the two individuals at crossing points. We calculated the absolute difference in time when the two participants had occupied a 2D position with minimum distance from the crossing points established during calibration. These asynchronies were averaged across trials and condition, separately for the four blocks to allow testing for improvement of coordination performance over time. ***Asynchrony*** was analysed with a repeated measures analysis of variance (rANOVA) with Block (4) and Coordination Demand (Congruent-Incongruent) as within-subjects factors.

***Mean Velocity (m/s)*** was computed as an individual measure of the speed of the movement between consecutive crossing points. Signed ***Spatial Deviation (cm)*** was computed as an individual measure of the spatial accuracy in tracing the given shape. This was calculated as the Euclidean distance between the actual and prescribed spatial position at each *corner* and at each midpoint of the *straight-line* segment between pairs of crossing points. Negative values indicate that the actual position was inside the prescribed shape and positive values indicate that it was outside of the prescribed shape. For the analyses of velocity and spatial deviation, we separately analysed segments of the trajectory that required a change in direction (*corners*) with segments that did not require a change in direction (the *straight-line* segments) because we expected that participants would modulate their actions in different ways when tracing corners and when tracing straight lines, especially on incongruent trials. We also included “Participant” (1 or 2) as a factor to see whether a leader–follower pattern emerged even though this was not instructed. Accordingly, ***Mean Velocity*** and ***Spatial Deviation*** were analysed with separate mixed analyses of variance (ANOVAs) with the factors Direction Change (2) and Coordination Demand (2) as within-subjects factors and Participant (2) as a between-subjects factor.

For all analyses, the significance level was set to an α level of 0.05. Significant interactions and main effects were analysed by Tukey post hoc tests.

### 2.2 Results

Only trials in which both participants waited for the go signal to start the movement, traced the correct shape, and performed four rounds of tracing were included in the analyses (mean % of trials discarded per pair = 3.3, range = 0–25%). For each dependent variable, participant and condition, we excluded as outliers values that fell three standard deviations above or below the mean (mean % of outliers per participant = 1.76, range = 1.19–2.47%).

#### 2.2.1 Asynchrony

Pairs were better synchronized in congruent (mean = 80 ms, SD = 20 ms) compared to incongruent trials (mean = 210 ms, SD = 40 ms), as shown by the significant main effect of Coordination Demand (*F*(1,9) = 84.56, *p* < 0.001, η^2^ = 0.90). Synchronization improved over time as confirmed by the significant main effect of Block (*F*(3,27) = 3.06, *p* = 0.04, η^2^ = 0.25) (mean and standard deviation of asynchrony per Block in Table 1). The interaction between Coordination Demand and Block was not significant (*p* = 0.70).

**Table 1:** Descriptives of the comparison of coordination performance (asynchrony) between Experiment 1 and Experiment 3 by means of a Bayesian independent *t*-test.

						95% Credible interval	
	Group	N	Mean	SD	SE	Lower	Upper
Main effect of experiment	Experiment 1	10	0.148	0.031	0.010	0.125	0.170
	Experiment 3	10	0.149	0.034	0.011	0.125	0.173
Congruency score	Experiment 1	10	−0.128	0.044	0.014	−0.159	−0.096
	Experiment 3	10	−0.143	0.070	0.022	−0.193	−0.092

#### 2.2.2 Mean velocity

**Fig. 3:**
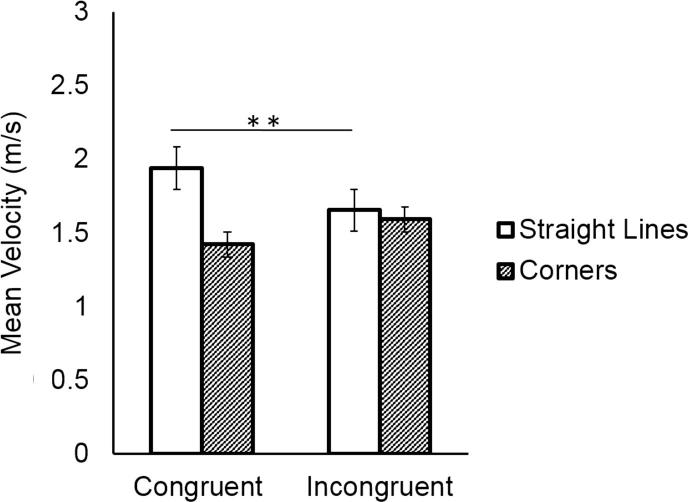
In the congruent condition, participants moved faster along straight-line segments than around corners. In the incongruent condition, they adjusted to their partner, slowing down on straight-line segments. Asterisks indicate the significance level of Tukey post hoc tests (^*^: *p* < 0.05, ^**^: *p* < 0.01).

Participants were faster when tracing the *straight-line* segments of the trajectory (mean = 2.09 m/s, SD = 0.48 m/s), and slowed down when tracing the *corner* segments which required a direction change (mean = 1.44 m/s, SD = 0.28 m/s), as shown by the significant main effect of Direction Change (*F*(1,18) = 97.45, *p* < 0.001, η^2^ = 0.84). Moreover, they were slower in incongruent trials (mean = 1.60, SD = 0.43) compared to congruent trials (mean = 1.93 m/s, SD = 0.53 m/s, main effect of Coordination Demand: *F*(1,18) = 42.21, *p* < 0.001, η^2^ = 0.70). The analysis showed a significant interaction of Direction Change and Coordination Demand (*F*(1,18) = 23.53, *p* < 0.001, η^2^ = 0.56), indicating that both participants slowed down in *straight-line* segments in incongruent trials compared to congruent trials (mean velocity of *straight-line* in congruent trials vs. *straight-line* in incongruent Trials: *p* = 0.0001), to the point that the average velocity of *straight-line* and *corner* segments in incongruent trials was not significantly different (*p* = 0.99). The analysis also showed a significant three-way interaction between Direction Change, Coordination Demand and Participant (*F*(1,18) = 5.21, *p* = 0.03, η^2^ = 0.22). However, post-hoc tests revealed that there were no significant between-subjects contrasts (all *p*s > 0.66). See Fig. 3.

#### 2.2.3 Spatial deviation

**Fig. 4:**
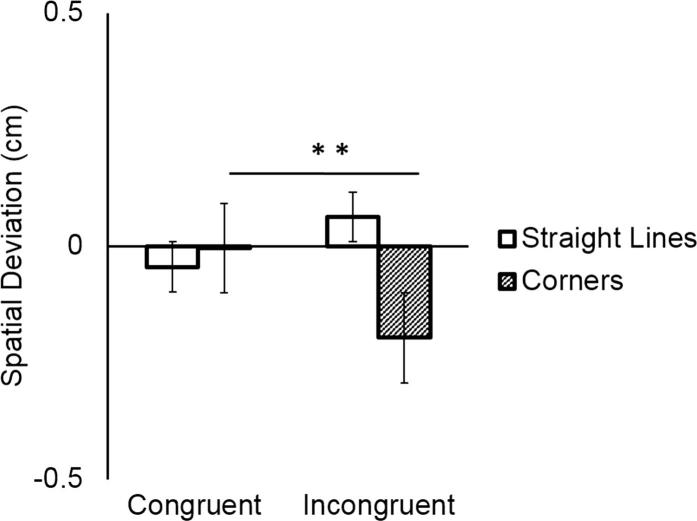
Participants deviated most from the prescribed shape at corners in the incongruent condition.

Participants deviated more from the prescribed shape when tracing *corner* segments (mean = −0.10 cm, SD = 0.21 cm) than when tracing *straight-line* segments (mean = 0.008 cm, SD = 0.11 cm), as shown by the significant main effect of Direction Change (*F*(1,18) = 5.10, *p* = 0.03, η^2^ = 0.22). As predicted, this deviation occurred specifically in incongruent trials where participants traded off spatial accuracy to coordinate with their partner. This was reflected in the significant Direction Change × Coordination Demand interaction (*F*(1,18) = 52.63, *p* = 0.000, η^2^ = 0.74), and the significant post hoc test (mean Spatial Deviation of *corner* in congruent trials vs. *corner* in incongruent trials: *p* = 0.001). Main effects and interactions with the factor Participant were not significant (all *p*s > 0.147). See Fig. 4.

### 2.3 Discussion

As expected, coordination was better in congruent than in incongruent trials, as reflected in lower mean absolute asynchrony. While coordination in congruent trials could be achieved by aligning the velocity of the movement to the same trajectory of the partner, incongruent trials required a trade-off between temporal and spatial coordination. How did pairs achieve coordination in incongruent trials? The results indicate that participants combined the temporal coordination strategy of matching the velocity profiles of their partner with the spatial coordination strategy of reducing the spatial differences between their own and the partner’s trajectory. Both participants slowed down in *straight-line* segments in incongruent compared to congruent trials to the point that the average velocity of *straight-line* and *corner* segments in incongruent trials was not significantly different. By doing so, they reduced the temporal incongruence between their movements. In addition, both participants “cut corners”, i.e. they deviated from the prescribed shape in the corner segments to reduce the incongruence between their movements.

The analysis comparing the two participants separately (participant 1, participant 2) showed no difference in their performance. There was no indication of an emergent leader-follower dynamic, as both participants adjusted temporally and spatially to their partner. This extends earlier findings (Konvalinka et al., 2010, Noy et al., 2011) by demonstrating that equal contributions to coordination occur not only when temporal and spatial coordination demands are congruent but also when they are incongruent, requiring a trade-off.

The results on asynchrony showed that pairs improved their temporal coordination over time. This indicates that the predictive models that participants relied on to generate predictions about each other’s actions became more accurate in the course of the interaction. It is unlikely that the observed coordination could have been achieved by implementing a reactive coordination strategy, where one makes adjustments after detecting that coordination is not going smoothly, as in this case individuals would constantly have been lagging behind one another. It is important to note that coordination in our task was not rhythmic, and was not fully determined by the shapes to be traced. In fact, to move in synchrony in Incongruent trials, individuals had to continuously adjust their velocity going against the natural velocity profile afforded by the shapes. Moreover, the spatial deviations from the determinist shape that we observed (participants cutting corners) worked against rhythmicity, therefore highlighting the importance of predictive mechanisms in solving a coordination task without a rhythmically-predictable structure.

One may argue that we cannot fully conclude that coupling of the internal predictive models of the two members in a pair was necessary for successful coordination. Was the mutual coupling of individual internal models necessary or would one partner predicting the other be sufficient for coordination? Experiment 2 was designed to address this question.

## 3 Experiment 2 – Reciprocal information flow with role assignment

In Experiment 2 we added a role manipulation: we instructed one participant to lead the interaction by establishing the tempo of the movements, and we instructed the other participant to adapt to the partner’s velocity in order to coordinate. We expected that this role assignment would keep the participant setting the tempo from adapting to and predicting their partner, allowing us to test whether one-way predictions are detrimental for coordination. On the one hand, prior research on emergent role distributions suggests that coordination can be very effective when one task partner leads the interaction and behaves very predictably, while the other anticipates and adjusts to the partner’s movements (Richardson et al., 2015, Skewes et al., 2015, Vesper et al., 2013). On the other hand, the incongruent condition in our task demands a complex trade-off between spatial and temporal coordination that might be more readily or perhaps even exclusively achieved by two individuals mutually predicting each other’s movements. If mutual adaptation (enabled by the coupling of two internal models) is necessary to solve the complex coordination trade-off, coordination in incongruent trials in Experiment 2 should be worse compared to Experiment 1.

### 3.1 Methods

#### 3.1.1 Participants

Ten randomly composed pairs of right-handed individuals (15 females, average age = 24.7 years, SD age = 2.9 years) participated in the study. The members in each pair did not know each other prior to participation. They signed prior informed consent and received monetary compensation. The study was performed in accordance with the Declaration of Helsinki.

#### 3.1.2 Apparatus

This was the same as in Experiment 1.

#### 3.1.3 Procedure

This was the same as in Experiment 1 with the following exceptions: At the beginning of the experiment participants were randomly assigned to the Leader and the Follower role. Each participant received different task instructions according to the assigned role. Leaders: “Trace your shape keeping the tempo you trained in the individual practice and be as spatially accurate as possible without stopping. In each trial you will hear “yellow” or “white”, instructing you to trace the yellow or white shape. Your partner will hear “same” or “different”. His/her task is to be coordinated with you, i.e., to meet you at the crossing points of the two shapes. In some trials you will trace the same shape, in some trials each of you will trace a different shape.” Followers: “Trace your shape being as spatially accurate as possible without stopping. Your task is to be coordinated with your partner, i.e., to meet your partner at the crossing points of the two shapes. At each trial you will hear “same” or “different”, instructing you to trace the same or the opposite shape your partner is tracing. Your partner will hear “yellow” or “white” and trace the corresponding shape.”

As in Experiment 1, participants received a tone as go signal (100 ms duration, 880 Hz). The participant in the Leader role was instructed to start and the participant in the Follower role started when the Leader reached the midline of the first segment of the shape.

#### 3.1.4 Data analyses

The same analyses described in Experiment 1 were employed in Experiment 2. Additionally, to compare coordination performance between the two experiments, we analysed *Asynchrony* with a mixed analysis of variance (ANOVA) with Coordination Demand (2) and Block (4) as within-subjects factors and Experiment (2) as a between-subjects factor. To compare individual movement parameters between experiments, we also performed an ANOVA on Mean Velocity and Spatial Deviation with Direction change (2) and Coordination Demand (2) as within-subjects factors and Experiment (2) as a between-subjects factor (see supplementary material).

### 3.2 Results

Only trials in which both participants waited for the go signal to start the movement, traced the correct shape, and performed four rounds of tracing were included in the analyses (mean % of trials discarded per pair = 2.5, range = 0–16%). For each dependent variable, participant and condition, we excluded as outliers the values that fell three standard deviations above or below the mean (mean % of outliers per participant = 1.34, range = 1.12–2.02%).

#### 3.2.1 Asynchrony

Pairs were better synchronized in congruent (mean = 70 ms, SD = 20 ms) compared to incongruent trials (mean = 280 ms, SD = 80 ms), as shown by the significant main effect of Coordination Demand (*F*(1,9) = 84.56, *p* < 0.001, η^2^ = 0.90). The analysis showed neither a significant main effect of Block (F(3,27) = 0.26, *p* = 0.85, η^2^ = 0.02), nor a significant interaction between Coordination Demand and Block (F(3,27) = 0.06, *p* = 0.97, η^2^ = 0.006).

#### 3.2.2 Mean velocity

**Fig. 5:**
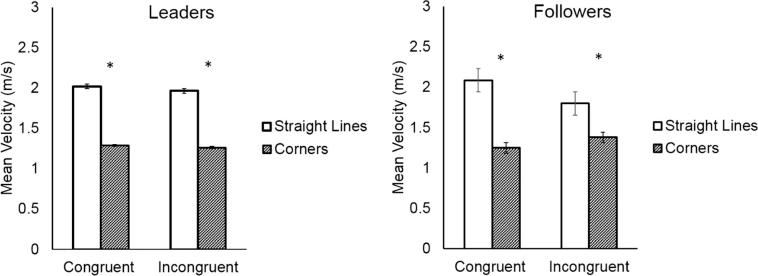
Participants’ mean velocity was higher during straight-line segments than during corners, regardless of the shape traced by their partner.

Participants were faster when tracing the *straight-line* segments of the shape (mean = 1.96 m/s, SD = 0.41 m/s), and slowed down when tracing the *corner* segments which required a direction change (mean = 1.29 m/s, SD = 0.23 m/s), as shown by the significant main effect of Direction Change (*F*(1,18) = 127.42, *p* < 0.001, η^2^ = 0.87). Moreover, they were slower in incongruent trials (mean = 1.60, SD = 0.43) compared to congruent trials (mean = 1.66 m/s, SD = 0.51 m/s) (main effect of Coordination Demand: *F*(1,18) = 13.48, *p* = 0.002, η^2^ = 0.42). See Fig. 5. The analysis also showed a significant interaction between Direction Change and Coordination Demand (*F*(1,18) = 4.66, *p* = 0.044, η^2^ = 0.20), but post hoc tests revealed no significant contrasts between conditions (all *p*s > 0.122).

#### 3.2.3 Spatial deviation

**Fig. 6:**
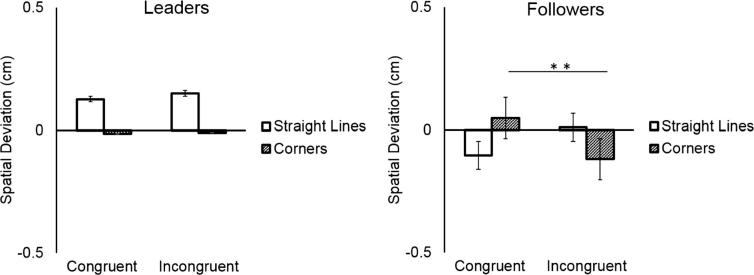
Whereas leaders did not change their movements depending on their partner’s shape, followers cut corners in incongruent trials.

Overall, Followers performed smaller movements (mean = -0.04 cm, SD = 0.16 cm) compared to Leaders (mean = 0.06 cm, SD = 0.17 cm), as shown by the significant main effect of Participant (*F*(1,18) = 5.33, *p* = 0.03, η^2^ = 0.23). As indicated by the significant interaction between Direction Change and Coordination Demand (*F*(1,18) = 15.98, *p* < 0.0001, η^2^ = 0.47), and the significant three-way interaction between Direction Change, Coordination Demand, and Participant (*F*(1,18) = 12.08, *p* = 0.002, η^2^ = 0.40), Followers systematically deviated from the prescribed shape in *corner* segments of incongruent trials where they traded off spatial accuracy to coordinate with their partner, compared to congruent trials where no spatial adaptation was required (mean Spatial Deviation of *corners* in congruent vs. incongruent trials: *p* < 0.01). Leaders, by contrast, did not alter their movements depending on the congruency of the trial (all *p*s > 0.98). See Fig. 6.

#### 3.2.4 Comparing coordination performance in Experiment 1 and Experiment 2

**Fig. 7:**
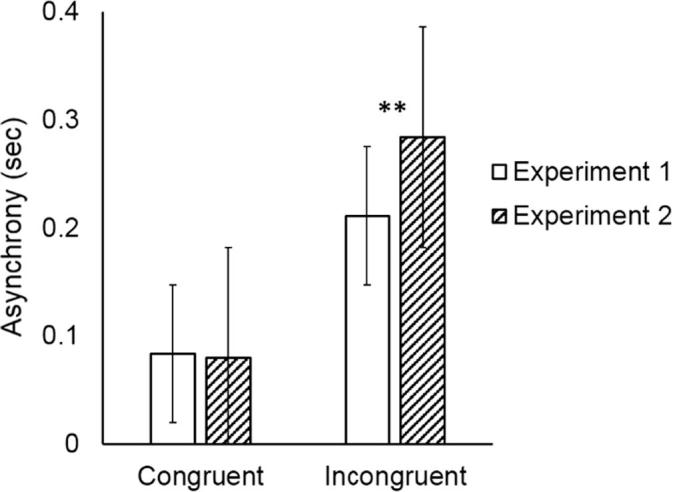
In the incongruent condition, pairs were more synchronized in Experiment 1 (reciprocal information flow without role assignment) than in Experiment 2 (reciprocal information flow with role assignment).

The results of the Block × Coordination Demand × Experiment mixed ANOVA indicate that overall pairs were better coordinated in congruent trials compared to incongruent ones (mean congruent trials: 81 ms, SD = 34 ms, mean incongruent trials: 247 ms, SD = 84 ms), as shown by the significant main effect of Coordination Demand (*F*(1,18) = 166.95, *p* < 0.0001, η^2^ = 0.90). Moreover pairs performing the task without role assignment (Experiment 1) were better coordinated in incongruent trials than pairs with role assignment (Experiment 2), as shown by the significant interaction between Coordination Demand and Experiment (*F*(1,18) = 8.89, *p* = 0.007, η^2^ = 0.33) and the significant post hoc test (mean incongruent trials in Experiment 1 vs. mean incongruent trials in Experiment 2: *p* = 0.01). All other *p*s > 0.086. See Fig. 7.

### 3.3 Discussion

Participants in Experiment 2 established better coordination in congruent than in incongruent trials and did not improve their coordination over time. They were significantly less coordinated in incongruent trials than pairs in Experiment 1 who performed the task without role assignment. This indicates that role assignment was detrimental for coordination, specifically when individuals needed to deal with incongruent coordination demands involving a complex trade-off between temporal and spatial dimensions of their movements.

Why did we observe these detriments in coordination in Experiment 2? In contrast to the results of Experiment 1, both participants failed to modulate their velocity profiles in an adaptive way, and therefore did not reduce the temporal incongruence of their movements. The results on spatial deviation show a clear effect of role distribution, where Followers “cut corners”, while Leaders did not alter their movements adaptively. The drop of coordination performance in Experiment 2 can be interpreted as a lack of mutual coupling of predictions: Leaders did not use the available perceptual information about the follower’s movements efficiently, as they focused on keeping the instructed tempo. Followers, on the other hand, did not perform optimally in their role as they failed to systematically adjust their velocity to the leaders’. Likely, the mutual availability of perceptual information created expectations in the Followers that the Leader would contribute to the coordination effort.

This raises the possibility that what disrupted coordination was not the role assignment per se but the combined availability of mutual feedback, which may have interfered with the implementation of role distribution. Being able to see each other’s movements may not only have made Followers less adaptive. It could also have affected Leader’s performance, as observing the Follower’s movements may have interfered with their own movements and kept them from being as predictable as possible. To test this explanation, we designed a third experiment where Leaders no longer had visual access to Followers’ movements.

## 4 Experiment 3 – Unidirectional information flow with role assignment

To investigate whether role assignment is generally detrimental for coordination, or only when combined with reciprocal information flow, we designed a third experiment in which we prevented mutual perceptual feedback. Participants were assigned to the role of Leader and Follower as in Experiment 2. However, Leaders did not have visual access to the movements of the Followers and were thus prevented from predicting and adapting to the partner’s movements. Introducing this manipulation allowed us to test whether a purely one-directional predictive model of interaction can sustain coordination. This could be the case if Leaders moved very predictably in the absence of visual information about their partner’s actions, thereby providing a stable input for the Follower’s predictive system.

### 4.1 Methods

#### 4.1.1 Participants

Ten randomly composed pairs of right-handed individuals (10 females, Average age = 25.4 years, SD age = 3.33 years) participated in the study. The members in each pair did not know each other prior to participation. They signed prior informed consent and received monetary compensation. The study was performed in accordance with the Declaration of Helsinki.

##### 4.1.1.1 Apparatus

This was the same as in Experiment 1 and 2, except that the panel between participants was a 50x50 cm one-way mirror (reflective on one side and see through on the other side). A set of 6 LED lamps was fixed on top of the frame and directed perpendicularly to the one-way mirror on the reflective side of it to ensure that the mirror was completely reflective on one side and completely see through on the other.

#### 4.1.2 Procedure

This was the same as in Experiment 2. The participant randomly assigned to the Leader role was sitting on the reflective side of the one-way mirror and could not see the Follower’s movements. The Follower was sitting on the see-through side of the mirror and could therefore see the Leader’s movements.

#### 4.1.3 Data analyses

The same analyses described in Experiment 2 were employed in Experiment 3. Additionally, to compare coordination performance to Experiment 1, we analysed *Asynchrony* with a mixed analysis of variance (ANOVA) with Coordination Demand (2) and Block (4) as within-subjects factors and Experiment (2) as a between-subjects factor. To compare individual movement parameters between experiments, we performed an ANOVA on Mean Velocity and Spatial Deviation with Direction change (2) and Coordination Demand (2) as within-subjects factors and Experiment (2) as a between-subjects factor (see supplementary material). To establish whether the predictability of Leaders’ movements was determined by the reciprocity of information flow we also performed a 2 × 3 mixed ANOVA with Experiment (3) as a between-subjects factor and Coordination Demand (2) as a within-subject factor on Leaders’ movement velocity and standard deviation (also reported in the supplementary material).

### 4.2 Results

Only trials in which both participants waited for the go signal to start the movement, traced the correct shape, and performed four rounds of tracing were included in the analyses (mean % of trials discarded per pair = 0.8, range = 0–8.3%). For each dependent variable, participant and condition, we excluded as outliers the values that fell three standard deviations above or below the mean (mean % of outliers per participant = 1.63, range = 1.12–2.22%).

#### 4.2.1 Asynchrony

Pairs were better synchronized in congruent (mean = 77 ms, SD = 30 ms) compared to incongruent trials (mean = 220 ms, SD = 60 ms), as indicated by the significant main effect of Coordination Demand (*F*(1,9) = 40.96, *p* < 0.001, η^2^ = 0.81). The analysis showed a trend towards significance of Block (F(3,27) = 2.31, *p* = 0.09, η^2^ = 0.20), indicating that pairs improved their coordination over time, but this did not reach statistical significance. There was no significant interaction between Coordination Demand and Block (F(3,27) = 0.68, *p* = 0.57, η^2^ = 0.07).

#### 4.2.2 Mean velocity

Participants were faster when tracing the *straight-line* segments of the shape (mean = 2.09 m/s, SD = 0.48 m/s), and slowed down when tracing the *corner* segments which required a direction change (mean = 1.44 m/s, SD = 0.28 m/s), as shown by the significant main effect of Direction Change (*F*(1,18) = 106.66, *p* < 0.0001, η^2^ = 0.85). Moreover, they were slower in incongruent trials (mean = 1.60 m/s, SD = 0.43 m/s) compared to congruent trials (mean = 1.93 m/s, SD = 0.53 m/s) (main effect of Coordination Demand: *F*(1,18) = 4.93, *p* = 0.03, η^2^ = 0.21).

**Fig. 8:**
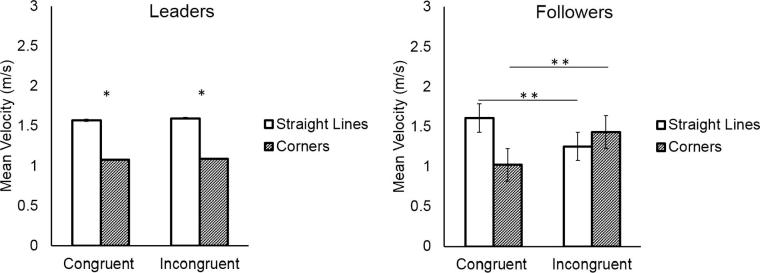
Whereas Leaders always moved faster during straight-line segments and slower during corners, followers in incongruent trials speeded up during corners and slowed down during straight-line segments.

The analysis showed a significant interaction between Direction Change and Participant (*F*(1,18) = 18.87, *p* < 0.001, η^2^ = 0.51), a significant interaction between Direction Change and Coordination Demand (*F*(1,18) = 27.72, *p* < 0.001, η^2^ = 0.60), and a significant three-way interaction between Direction Change, Coordination Demand, and Participant (*F*(1,18) = 30.37, *p* < 0.0001, η^2^ = 0.62). The results reveal that Followers slowed down in *straight-line* segments in incongruent trials compared to congruent trials (mean velocity of *straight-line* in congruent trials vs. *straight-line* in incongruent trials: *p* < 0.01), and they speeded up in *corner* segments in incongruent trials compared to congruent trials (mean velocity of *corner* in congruent trials vs. *corner* in incongruent trials: *p* < 0.001). This was not the case for Leaders, who did not alter their movements’ velocity depending on the congruency of the partner’s shape (all *p*s > 0.99). See Fig. 8.

#### 4.2.3 Spatial deviation

**Fig. 9:**
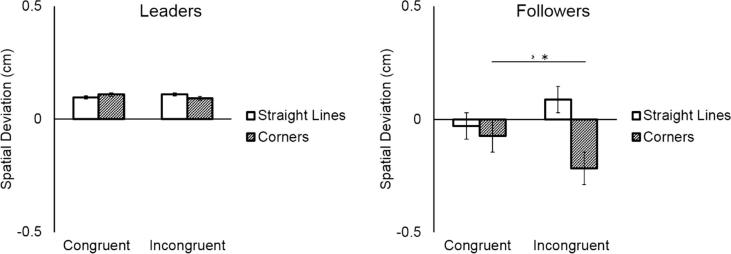
While Leaders did not modulate their trajectories, Followers cut corners in incongruent trials.

Overall Followers’ movement trajectories were less expansive (mean = -0.057 cm, SD = 0.19 cm) than Leaders’ (mean = 0.10 cm, SD = 0.19 cm), as shown by the significant main effect of Participant (*F*(1,18) = 8.11, *p* = 0.01, η^2^ = 0.31). As indicated by the significant interaction between Direction Change and Coordination Demand (*F*(1,18) = 7.40, *p* = 0.01, η^2^ = 0.29), and the three-way interaction between Direction Change, Coordination Demand, and Participant (*F*(1,18) = 4.63, *p* = 0.04, η^2^ = 0.20), Followers deviated from the prescribed shape in *corner* segments in incongruent trials (“cutting corners”), where they had to trade off spatial accuracy to coordinate with their partner, compared to congruent trials, where no spatial adaptation was required (mean Spatial Deviation of *corners* in congruent vs. incongruent trials: *p* < 0.001). Leaders, on the contrary, did not alter their movements according to the congruency of the trial (all *p*s > 0.99). See Fig. 9.

#### 4.2.4 Comparing coordination performance in Experiment 1 and Experiment 3

The results of the Block × Coordination Demand × Experiment mixed ANOVA indicate that overall pairs were better coordinated in congruent trials (mean = 0.080 ms, SD = 0.038 ms) compared to incongruent trials (mean = 0.215 ms, SD = 0.073 ms), as shown by the significant main effect of Coordination Demand (*F*(1,18) = 105.95, *p* < 0.0001, η^2^ = 0.85). Moreover, participants improved their coordination performance over time, as shown by the significant main effect of Block (*F*(3,54) = 4.65, *p* = 0.005, η^2^ = 0.20). The analysis failed to show a significant main effect of Experiment (*F*(1,18) = 0.011, *p* = 0.91, η^2^ = 0.00). Neither the interaction of Coordination Demand and Experiment was significant (*F*(1,18) = 0.32, *p* = 0.53, η^2^ = 0.01), nor the interaction of Block and Experiment (*F*(3,54) = 0.059, *p* = 0.62, η^2^ = 0.03), indicating that pairs in Experiment 1 (Reciprocal information flow without role assignment) and Experiment 3 (Unidirectional information flow with role assignment) achieved the same level of coordination performance. All other *p*s > 0.97.

In order to assess whether coordination performance was comparable in Experiment 1 and Experiment 3, we performed a Bayesian analysis using JASP (JASP Team, 2016). We computed an index of coordination performance for Experiment 1 and Experiment 3 averaging all trials and all conditions. Furthermore, we computed a Congruency score, as an index of the average difference between Congruent and Incongruent trials separately per each participant (this corresponds to the Congruency × Experiment comparison tested with the mixed ANOVA). On both indices separately, we performed a Bayesian independent *t*-test with a default uniform prior (0.707), testing the hypotheses that H1: coordination performance in Experiment 1 ≠ coordination performance in Experiment 3; H0: coordination performance in Experiment 1 = coordination performance in Experiment 3. These analyses resulted in Bayes Factors_01_ of 2.506 and 2.240, for the two analyses respectively (see Table 1 for descriptives). The results can be interpreted as the data being 2.5 (and 2.24) more likely under the null hypothesis (coordination performance is equal in Exp1 and Exp3). However, results need to be interpreted cautiously as they fail to provide strong evidence (Jeffreys, 1961). This indicates that further investigation is needed in order to corroborate this interpretation.

### 4.3 Discussion

As expected given the unidirectional information flow, Leaders did not adapt to Followers and kept their performance constant across conditions. Followers systematically modulated their velocity profile in incongruent trials to match their partners’ movements, speeding up at corners and slowing down during straight-line segments. They also adapted their trajectories, “cutting corners” to synchronize with the leaders’ movements. Importantly, when comparing coordination performance in terms of mean asynchrony between Experiment 1 and Experiment 3 we found no differences between experiments. However, when further investigating the absence of difference between the experiments with Bayesian statistics, we failed to find strong evidence. These results indicate that further investigation is required to corroborate the claim that no difference in performance was observed between experiments. Altogether, results suggest that Followers could exploit the visuo-motor information provided by the unresponsive but predictable Leaders to form accurate predictions of the observed actions (Wolpert & Kawato, 2003).

## 5 General discussion

Our findings demonstrate that in a complex task involving trade-offs between spatial and temporal coordination, two different ways of interacting with each other can lead to successful joint performance. Participants achieved high synchronization and improved their performance over time when they interacted without role assignment and could mutually perceive each other (Experiment 1). This indicates that reciprocal information flow allowed partners to mutually adapt to and predict each other’s actions, supporting the hypothesis that coupled internal models benefit coordination (Noy et al., 2011).

These results extend previous research by showing that mutual prediction is not only relevant for joint action tasks with congruent coordination demands (Konvalinka et al., 2010, Noy et al., 2011) but also facilitates coordination when spatial and temporal aspects need to be traded off. Furthermore, the results of Experiment 1 indicate that mutual alignment of velocity in the absence of a leader-follower dynamic may be more wide-spread than previously thought. In Noy and colleague’s study, people without experience in improvisation coordinating their actions in the context of reciprocal information flow could not help falling into a leader-follower dynamic and had difficulties aligning their velocity profiles. Our findings suggest that people without training in improvisation can actually align their velocity profiles by predicting each other’s actions in coordination tasks that do not involve improvisation.

A second way in which coordination can be achieved is by having a clear role distribution, but only in combination with environmental conditions that support the implementation of this role distribution (Experiment 3). Pairs in Experiment 3, where only one partner could perceive and adjust to the other’s actions, achieved similar levels of coordination as pairs in Experiment 1, where both partners mutually adjusted to each other. This suggests that Followers could exploit the visuo-motor information provided by the unresponsive but predictable Leaders to form accurate predictions of the observed actions. At first glance, this finding seems to be at odds with results reported by Konvalinka and colleagues (2010) where task partners performing a joint tapping task were more synchronized under reciprocal than under unidirectional information flow. However, Followers in our study had continuous access to the Leader’s movements, which likely facilitated prediction. In fact, Konvalinka et al. found that unidirectional coordination with a stable but unresponsive computer was as good as reciprocal coordination. Our results demonstrate that in a continuous visuo-motor coordination task human interaction partners can achieve the same level of coordination in a unidirectional and in a reciprocal set-up.

As demonstrated by Experiment 2, coordination on incongruent trials suffered when both partners could perceive each other’s actions but were assigned roles of Leader and Follower. We conclude that what disrupted coordination was not the role assignment per se but the concurrent availability of mutual feedback, which may have interfered with the implementation of role distribution. On the one hand, Leaders’ perception of Followers’ movements may have created interference, thereby making their actions less predictable (see supplementary material for the comparative analyses of leaders’ velocity and variability across experiments, confirming that Leaders in Experiment 2 were less predictable); on the other hand, Followers may not have made enough of an effort to coordinate with Leaders, expecting them to adjust despite their assigned role. While Noy and colleagues reported detrimental effects of role assignment under reciprocal information flow only in improvisation experts (2011), our findings indicate that negative effects of role assignment on coordination may be more wide-spread. Given prior evidence for the benefits of emergent role distribution (Richardson et al., 2015, Vesper et al., 2013), an interesting question for future research is how emergent and assigned role distributions relate to each other. For instance, in the present task role differentiation was not strictly required, whereas Richardson and colleagues’ task (2015) necessitated role distribution. It is possible that a strong need for role differentiation eliminates or reduces any negative effects of mutual feedback. This could be tested by investigating how followers’ behavior changes as a consequence of their belief about leaders’ ability to adapt to them, or their knowledge about instructions given to leaders’.

Finally, it is important to note that role assignment in the context of reciprocal information flow specifically affected incongruent trials that involved a trade-off between spatial and temporal coordination. As the congruent task demands were much easier to deal with, synchronization on congruent trials was high across all three experiments, confirming that performance in these trials is not predictive of coordination when temporal and spatial dimensions of co-actors’ movements are incongruent. This reveals that coordination tasks involving trade-offs between spatial and temporal aspects are especially important for understanding how social context affects performance limits in joint action. Indeed, many of the joint actions we engage in cannot be performed without balancing different coordination demands. The present study reveals that trade-offs between spatial and temporal aspects of coordination can be managed both by mutually predicting and adjusting to each other’s actions, and by following a clear task distribution in terms of Leader-Follower. Questions for future research include whether some forms of coordination can only be achieved through mutual prediction and adaptation, and how interaction partners deal with other trade-offs, such as achieving high speed versus high accuracy.

## Appendix A Supplementary material

Supplementary Data S2

This is open data under the CC BY license (http://creativecommons.org/licenses/by/4.0/).

Supplementary Data S3

This is open data under the CC BY license (http://creativecommons.org/licenses/by/4.0/).

Supplementary Data S4

This is open data under the CC BY license (http://creativecommons.org/licenses/by/4.0/).

## References

[b0005] Flash T., Hogan N. (1985). The coordination of arm movements: An experimentally confirmed mathematical model. Journal of Neuroscience.

[b0010] Franklin D.W., Wolpert D.M. (2011). Computational mechanisms of sensorimotor control. Neuron.

[b0020] Jeffreys H. (1961). Theory of probability.

[b0025] Kawato M. (1999). Internal models for motor control and trajectory planning. Current Opinion in Neurobiology.

[b0030] Keller P.E., Knoblich G., Repp B.H. (2007). Pianists duet better when they play with themselves: On the possible role of action simulation in synchronization. Consciousness and Cognition.

[b0035] Keller P.E., Novembre G., Hove M.J. (2014). Rhythm in joint action: Psychological and neurophysiological mechanisms for real-time interpersonal coordination. Philosophical Transactions of the Royal Society B.

[b0040] Knoblich G., Jordan J.S. (2003). Action coordination in groups and individuals: Learning anticipatory control. Journal of Experimental Psychology: Learning, Memory, and Cognition.

[b0045] Konvalinka I., Vuust P., Roepstorff A., Frith C.D. (2010). Follow you, follow me: Continuous mutual prediction and adaptation in joint tapping. The Quarterly Journal of Experimental Psychology.

[b0050] Kourtis D., Sebanz N., Knoblich G. (2013). Predictive representation of other people's actions in joint action planning: An EEG study. Social Neuroscience.

[b0060] Neilson P.D., Neilson M.D., O'Dwyer N.J. (1988). Biological Cybernetics.

[b0065] Noy L., Dekel E., Alon U. (2011). The mirror game as a paradigm for studying the dynamics of two people improvising motion together. Proceedings of the National Academy of Sciences.

[b0070] Ramenzoni V.C., Sebanz N., Knoblich G. (2015). Synchronous imitation of continuous action sequences: The role of spatial and topological mapping. Journal of Experimental Psychology: Human Perception and Performance.

[b0075] Richardson M.J., Harrison S.J., Kallen R.W., Walton A., Eiler B.A., Saltzman E., Schmidt R.C. (2015). Self-organized complementary joint action: Behavioral dynamics of an interpersonal collision-avoidance task. Journal of Experimental Psychology: Human Perception and Performance.

[b0080] Richardson M.J., Campbell W.L., Schmidt R.C. (2009). Movement interference during action observation as emergent coordination. Neuroscience Letters.

[b0085] Roberts M.E., Goldstone R.L. (2011). Adaptive group coordination and role differentiation. PLoS One.

[b0095] Skewes J.C., Skewes L., Michael J., Konvalinka I. (2015). Synchronised and complementary coordination mechanisms in an asymmetric joint aiming task. Experimental Brain Research.

[b0100] Vesper C., Schmitz L., Safra L., Sebanz N., Knoblich G. (2016). The role of shared visual information for joint action coordination. Cognition.

[b0105] Vesper C., van der Wel R.P., Knoblich G., Sebanz N. (2011). Making oneself predictable: Reduced temporal variability facilitates joint action coordination. Experimental Brain Research.

[b0110] Vesper C., van der Wel R.P., Knoblich G., Sebanz N. (2013). Are you ready to jump? Predictive mechanisms in interpersonal coordination. Journal of Experimental Psychology: Human Perception and Performance.

[b0115] Vuust P., Witek M.A. (2014). Rhythmic complexity and predictive coding: a novel approach to modeling rhythm and meter perception in music. Frontiers in Psychology.

[b0120] Wolpert D.M., Doya K., Kawato M. (2003). A unifying computational framework for motor control and social interaction. Philosophical Transactions of the Royal Society of London B: Biological Sciences.

