# Supplementary material for: Reciprocal information flow and role distribution support joint action coordination
Source: Cognition. 2019 Jun;187:21–31. doi: 10.1016/j.cognition.2019.02.006 (PMC6446186; doi:10.1016/j.cognition.2019.02.006)
Supplement: Supplementary Data 1 [file mmc1.docx]

**Supplementary Analyses**

**1. Comparing Leaders’ Velocity across Experiments**

Here, we compared Leaders’ movement velocity and standard deviation between all three experiments to establish whether the predictability of Leaders’ movements was determined by the reciprocity of information flow (2 x 3 mixed ANOVA with Experiment (3) as a between-subjects factor and Coordination Demand (2) as a within-subject factor).

The results of the Coordination Demand x Experiment mixed ANOVA on Mean Velocity indicate that Leaders in Experiment 3 (Unidirectional information flow with role assignment) were slower compared to both leaders in Experiment 1 (Reciprocal information flow without role assignment) and in Experiment 2 (Reciprocal information flow with role assignment) (*p* = .0006 and *p* = .01, respectively), as shown by the significant main effect of Experiment (*F*(1,27) = 9.72, *p* = .0006, η^2^ = 0.42). Moreover, the analysis showed a significant main effect of Coordination Demand (*F*(1,27) = 25.68, *p* < .0001, η^2^ = 0.48), and a significant interaction of Coordination Demand and Experiment (*F*(1,27) = 21.10, *p* < .0001, η^2^ = 0.60), indicating that Leaders in Experiment 1 (Reciprocal information flow without role assignment) where faster in Congruent trials compared to the other experiments (all *p*s < .037).

The results of the Coordination Demand x Experiment mixed ANOVA on Standard Deviation of Velocity indicate that Leaders in Experiment 3 (Unidirectional information flow with role assignment) were less variable compared to both leaders in Experiment 1 and Experiment 2 (*p* = .0001 and *p* = .04, respectively), as reflected in the significant main effect of Experiment (*F*(1,27) = 13.09, *p* = .0001, η^2^ = 0.49). Neither the main effect of Coordination Demand was significant (*F*(1,27) = 2.14, *p* = .15, η^2^ = 0.07), nor the interaction of Coordination Demand and Experiment (*F*(1,27) = 0.088, *p* = .42, η^2^ = 0.06).

***2. Comparing individual movement parameters in Experiment 1 and Experiment 2***

Here we report the comparison across experiments of individual movement parameters by means of a mixed analysis of variance (ANOVA) with Direction change (2) and Coordination Demand (2) as within-subjects factors, and Experiment (2) as a between-subjects factor for Participant 1 and Participant 2 (Experiment 1), and Leader and Followers respectively (Experiment 2).

*2.1 Mean Velocity (Participants 1 vs. Leaders)*

The results of the Direction Change x Coordination Demand x Experiment mixed ANOVA indicate that Participants 1 were faster in congruent compared to incongruent trials both when performing straight line and corner segments, as shown by the interaction of Direction Change x Coordination Demand x Experiment (*F*(1,18) = 17.36, *p* = 0.0005, η^2^ = 0.49) and the significant post hoc tests (all *p*s < 0.012). The analyses showed significant main effects of Coordination Demand (*F*(1,18) = 33.96, *p* = 0.0002, η^2^ = 0.65) and of Direction Change (*F*(1,18) = 155.39, *p* < 0.0001, η^2^ = 0.89), a significant Coordination Demand x Experiment interaction (*F*(1,18) = 20.776, *p* = 0.0002, η^2^ = 0.53) and a significant Direction Change x Coordination Demand interaction (*F*(1,18) = 23.317, *p* = 0.0001, η^2^ = 0.56).

*2.2 Mean Velocity (Participants 2 vs. Followers)*

The results of the Direction Change x Coordination Demand x Experiment mixed ANOVA indicate that Participants 2 were faster in congruent compared to incongruent trials, as shown by the significant interaction between Coordination Demand x Experiment (*F*(1,18) = 8.13, *p* = 0.01, η^2^ = 0.31) and the significant post hoc test (mean velocity in incongruent trials vs. mean velocity in congruent trials of Participants 2: *p* < 0.001; all other *p*s > 0.14). The analyses showed significant main effects of Coordination Demand (*F*(1,18) = 22.43, *p* = 0.0001, η^2^ = 0.55) and of Direction Change (*F*(1,18) = 78.45, *p* < 0.0001, η^2^ = 0.81), and a significant Coordination Demand x Direction Change interaction (*F*(1,18) = 17.51, *p* < 0.001, η^2^ = 0.49).

*2.3 Spatial Deviation (Participants 1 vs. Leaders)*

The results of the Direction Change x Coordination Demand x Experiment mixed ANOVA indicate that Participants 1 were less spatially accurate than Leaders, as shown by the main effect of Experiment (*F*(1,18) = 7.67, *p* = 0.01, η^2^ = 0.29). This result indicates that Participants 1 reduced the overall trajectory they performed (mean Spatial Deviation of Participants 1 = -0.04), while Leaders did not (mean Spatial Deviation of Leaders = 0.06). The analyses showed a significant Direction Change x Coordination Demand (*F*(1,18) = 30.77, *p* < 0.0001, η^2^ = 0.63) and a significant Direction Change x Coordination Demand x Experiment interaction (*F*(1,18) = 22.02, *p* = 0.0001, η^2^ = 0.55). The latter indicates that Participants 1 reduced their trajectory in incongruent trials by “cutting corners” (mean Spatial Deviation of Corners in incongruent trials vs. mean Spatial Deviation of Corners in congruent trials: p = 0.0002). On the contrary, Leaders did not alter their trajectory with respect to the congruency of the coordination demand (mean Spatial Deviation of Corners in incongruent trials vs. mean Spatial Deviation of Corners in congruent trials: p = 1.000).

*2.4 Spatial Deviation (Participants 2 vs. Followers)*

The results of the Direction Change x Coordination Demand x Experiment mixed ANOVA indicate that both Participants 2 and Followers reduced their trajectory in incongruent trials by “cutting corners”, as shown by the significant Direction Change x Coordination Demand interaction (*F*(1,18) = 39.97, *p* < 0.0001, η^2^ = 0.68) and the significant post hoc test (mean Spatial Deviation of Corners in incongruent trials vs. mean Spatial Deviation of Corners in congruent trials: p = 0.0003). The analyses failed to show a main effect of Experiment (*F*(1,18) = 0.02, *p* = 0.50, η^2^ = 0.02), or other significant interactions, suggesting no difference in movement parameters between Participant 2 and Followers. All other *p*s > 0.18.

***3. Comparing individual movement parameters in Experiment 1 and Experiment 3***

Here we report the comparison across experiments of individual movement parameters by means of a mixed analysis of variance (ANOVA) with Direction change (2) and Coordination Demand (2) as within-subjects factors, and Experiment (2) as a between-subjects factor separately for Participant 1 and Participant 2 (Experiment 1), and Leader and Followers respectively (Experiment 3).

*3.1 Mean Velocity (Participants 1 vs. Leaders)*

The results of the Direction Change x Coordination Demand x Experiment mixed ANOVA indicate that Participants 1 were faster than Leaders in congruent compared to incongruent trials both when performing straight line and corner segments, as shown by the significant interaction between Direction Change x Coordination Demand x Experiment (*F*(1,18) = 17.36, *p* = 0.0005, η^2^ = 0.49) and the significant post hoc tests (all *p*s < 0.012). The analysis also showed significant main effects of Experiment (*F*(1,18) = 25.14, *p* < 0.0001, η^2^ = 0.58), Coordination Demand (*F*(1,18) = 25.13, *p* < 0.0001, η^2^ = 0.58) and of Direction Change (*F*(1,18) = 191.65, *p* < 0.0001, η^2^ = 0.91), as well as significant interactions between Direction Change x Experiment (*F*(1,18) = 7.75, *p* = 0.01, η^2^ = 0.30), Coordination Demand x Group (*F*(1,18) = 18.66, *p* = 0.0004, η^2^ = 0.50) and Direction Change x Coordination Demand (*F*(1,18) = 22.72, *p* = 0.0001, η^2^ = 0.55).

*3.2 Mean Velocity (Participants 2 vs. Followers)*

The results of the Direction Change x Coordination Demand x Experiment mixed ANOVA indicate that Participants 2 were faster than Followers, as shown by the main effect of Experiment (*F*(1,18) = 34.51, *p* < 0.0001, η^2^ = 0.65), and this was true for both congruent and incongruent trials (Coordination Demand x Experiment significant interaction (*F*(1,18) = 8.13, *p* = 0.01, η^2^ = 0.31, all *p*s < 0.01). The analyses showed a significant Direction Change x Experiment interaction (*F*(1,18) = 8.69, *p* = 0.008, η^2^ = 0.32), indicating that Participants 2 were faster than Followers both when tracking straight-line and corner segments of the trajectory (post hoc tests: all *p*s < 0.03). The analysis also showed significant main effects of Coordination Demand (*F*(1,18) = 12.58, *p* = 0.002, η^2^ = 0.41) and of Direction Change (*F*(1,18) = 41.05, *p* < 0.0001, η^2^ = 0.69), and a significant Coordination Demand x Direction Change interaction (*F*(1,18) = 34.14, *p* < 0.0001, η^2^ = 0.65).

*3.3 Spatial Deviation (Participants 1 vs. Leaders)*

The results of the Direction Change x Coordination Demand x Experiment mixed ANOVA indicate that Participants 1 were less spatially accurate than Leaders, as shown by the main effect of Experiment (*F*(1,18) = 8.28, *p* = 0.01, η^2^ = 0.31). This result indicates that Participants 1 reduced the overall trajectory they performed (mean Spatial Deviation of Participants 1 = -0.04 cm), while Leaders did not (mean Spatial Deviation of Leaders = 0.10 cm). The analyses showed a significant interaction between Direction Change x Coordination Demand (*F*(1,18) = 33.71, *p* < 0.0001, η^2^ = 0.65) and a significant interaction between Direction Change x Coordination Demand x Experiment (*F*(1,18) = 20.10, *p* = 0.0002, η^2^ = 0.52). The latter indicates that Participants 1 reduced their trajectory in incongruent trials by “cutting corners” (mean Spatial Deviation of Corners in incongruent trials vs. mean Spatial Deviation of Corners in congruent trials: *p* = 0.0002). On the contrary, Leaders did not alter their trajectory with respect to the congruency of Coordination Demand (mean Spatial Deviation of Corners in incongruent trials vs. mean Spatial Deviation of Corners in congruent trials: *p* = 1.000). All other *p*s > 0.11.

*3.4 Spatial Deviation (Participants 2 vs. Followers)*

The results of the Direction Change x Coordination Demand x Experiment mixed ANOVA indicate that both Participants 2 and Followers reduced their trajectory in incongruent trials by “cutting corners”, as shown by the significant Direction Change x Coordination Demand interaction (*F*(1,18) = 23.98, *p* = 0.0001, η^2^ = 0.57) and the significant post hoc test (mean Spatial Deviation of Corners in incongruent trials vs. mean Spatial Deviation of Corners in congruent trials: *p* = 0.003). The analyses failed to show a main effect of Experiment (*F*(1,18) = 0.02, *p* = 0.86, η^2^ = 0.01), or other significant interactions, suggesting no difference in movement parameters between Participant 2 and Followers. All other *p*s > 0.33.

***4. Comparing individual movement parameters in Experiment 2 and Experiment 3***

Here we report the comparison across experiments of individual movement parameters by means of a mixed analysis of variance (ANOVA) with Direction change (2) and Coordination Demand (2) as within-subjects factors, and Experiment (2) as a between-subjects factor separately for Leaders (Experiment 2 vs. Experiment 3), and Followers (Experiment 2 vs. Experiment 3).

*4.1 Mean Velocity of Leaders (Experiment 2 vs. Experiment 3)*

The results of the Direction Change x Coordination Demand x Experiment mixed ANOVA indicate that Leaders in Experiment 2 were faster than Leaders in Experiment 3 in both congruent and incongruent trials both when performing straight line and corner segments, as shown by the significant interaction between Direction Change x Coordination Demand x Experiment (*F*(1,18) = 10.92, *p* = 0.004, η^2^ = 0.37). The analysis also showed significant main effects of Experiment (*F*(1,18) = 8.41, *p* < 0.01, η^2^ = 0.32), and of Direction Change (*F*(1,18) = 138.98, *p* < 0.001, η^2^ = 0.86), as well as significant interactions between Direction Change x Experiment (*F*(1,18) = 4.55, *p* = 0.047, η^2^ = 0.03), and Coordination Demand x Group (*F*(1,18) = 14.20, *p* = 0.001, η^2^ = 0.41).

*4.2 Mean Velocity of Followers (Experiment 2 vs. Experiment 3)*

The results of the Direction Change x Coordination Demand x Experiment mixed ANOVA indicate that Followers in Experiment 2 were faster than Followers in Experiment 3 in both congruent and incongruent trials, as shown by the significant interaction between Coordination Demand x Experiment (*F*(1,18) = 9.06, *p* = 0.008, η^2^ = 0.31) and Followers in Experiment 2 were faster than Followers in Experiment 3 when performing straight lines, as shown by the significant interaction between Direction Change x Experiment (*F*(1,18) = 22.05, *p* < 0.001, η^2^ = 0.177).The analysis also showed significant main effects of Experiment (*F*(1,18) = 9.24, *p* = 0.007, η^2^ = 0.33), and of Direction Change (*F*(1,18) = 84.37, *p* < 0.001, η^2^ = 0.67), as well as significant interactions between Direction Change x Coordination Demand (*F*(1,18) = 22.62, *p* < 0.001, η^2^ = 0.53).

*4.3 Spatial Deviation of Leaders (Experiment 2 vs. Experiment 3)*

The results of the Direction Change x Coordination Demand x Experiment mixed ANOVA showed a significant interaction between Direction Change x Coordination Demand (*F*(1,18) = 6.28, *p* < 0.02, η^2^ = 0.25), indicating that overall Leaders had a larger Spatial Deviation in Lines compared to Corners in both congruent and incongruent trials. The analyses failed to show a main effect of Experiment (*F*(1,18) = 0.47, *p* = 0.49, η^2^ = 0.02), or other significant interactions, suggesting no difference in movement parameters between Leaders in the two experiments. All other *p*s > 023.

*4.4 Spatial Deviation of Followers (Experiment 2 vs. Experiment 3)*

The results of the Direction Change x Coordination Demand x Experiment mixed ANOVA indicate that Followers of both experiments reduced their trajectory in incongruent trials by “cutting corners”, as shown by the significant Direction Change x Coordination Demand interaction (*F*(1,18) = 17.59, *p* = 0.0005, η^2^ = 0.49) and the significant post hoc test (mean Spatial Deviation of Corners in incongruent trials vs. mean Spatial Deviation of Corners in congruent trials: *p* = 0.0009). The analyses showed also a significant main effect of Direction Change (*F*(1,18) = 4.68, *p* = 0.044, η^2^ = 0.20), and a Direction Change x Experiment significant interaction (*F*(1,18) = 6.06, *p* = 0.02, η^2^ = 0.25). However post-hoc analysis showed no significant differences in the between experiments contrasts. All other *p*s > 0.34.
